# Comment on: Combined protocol for severe and moderate acute malnutrition in emergencies: Stakeholder perspectives in four countries

**DOI:** 10.1111/mcn.13021

**Published:** 2020-06-24

**Authors:** Jessica Bourdaire, Pramila Ghimire, Carrie Morrison, Benedict Tabiojongmbeng, Mona Shaikh, Dina Aburmishan, Hussein Mahad, Nicolas Joannic, Jennifer Rosenzweig

**Affiliations:** ^1^ Nutrition Specialist, United Nations World Food Programme Rome Italy; ^2^ Head of Nutrition, United Nations World Food Programme Mogadishu Somalia; ^3^ Head of Nutrition, United Nations World Food Programme Abuja Nigeria; ^4^ Head of Nutrition, United Nations World Food Programme Niamey Niger; ^5^ Head of Nutrition, United Nations World Food Programme Juba South Sudan; ^6^ Deputy Head of Nutrition, United Nations World Food Programme Juba South Sudan; ^7^ Nutrition Officer, United Nations World Food Programme Juba South Sudan; ^8^ Chief Nutrition Operations, United Nations World Food Programme Rome Italy; ^9^ Chief Knowledge Management and Digital Innovation, United Nations World Food Programme Rome Italy

The World Food Programme (WFP) remains committed to exploring new modalities to improve early detection and treatment coverage for moderate acute malnutrition (MAM) and severe acute malnutrition (SAM) and, more importantly, to prevent malnutrition altogether.

The article contains various wrong assertions about WFP nutrition programming. First, WFP Nigeria has not experienced any budget reduction for MAM treatment in recent years. Equally, WFP South Sudan does not have restrictions to subcontract monitoring. Incidentally, ready‐to‐use supplementary food (RUSF) is not the only food supplement utilised by WFP for treating MAM children. RUSF and Super Cereal Plus are used in Niger, Nigeria and South Sudan. Similarly, in Nigeria, where targeted supplementary feeding programmes (TSFP) are not included in the national protocol, WFP has been treating MAM children using RUSF and Super Cereal Plus through blanket supplementary feeding programme (BSFP) platforms since 2017 in conflict affected areas.

This article analyses the perspectives of stakeholders interviewed by the authors, affiliated with IRC, about the Combined Protocol which utilises ready‐to‐use therapeutic food (RUTF). Availability of RUSF was used as a proxy indicator of RUSF pipeline reliability. However, the use of Super Cereal Plus for MAM children and the provision of MAM treatment through BSFP, both related to RUSF availability, were overlooked. To assess RUSF and RUTF pipeline reliability, considering the increased caseload resulting from treating SAM and MAM with one product, the authors should have examined supply chain elements such as last mile delivery and volume, since worldwide MAM children outnumber by far SAM children.

